# Supplementary material for: Multicriteria global optimization for biocircuit design
Source: BMC Syst Biol. 2014 Sep 24;8:113. doi: 10.1186/s12918-014-0113-3 (PMC4180256; doi:10.1186/s12918-014-0113-3)
Supplement: Additional file 1 — Model equations and parameters. Model equations and parameters corresponding to the example. [file 12918_2014_113_MOESM1_ESM.pdf]

Additional File 1.

Multicriteria global optimization for biocircuit design

Irene Otero-Muras and Julio R. Banga

Bioprocess Engineering Group, Spanish Council for Scientific Research, IIM-CSIC, Eduardo Cabello 6, 36208 Vigo (Spain)

This additional file contains the generic equations for the model of the genetic circuit used as a working example through the main text. The dynamic model equations and the kinetic parameters have been obtained from Ref. [1].

Model Equations and Parameters

The states of the generic model are the concentrations of all the species involved $lacI$, $LacIIPTG$, $tetR$, $tetRaTc$, cI, $araC$ and the generic model consists of 6 ordinary differential equations representing the mass balances for the system. The coefficients $Y(i,j)$ for $i = 1,\dots,8$ and $j = 1,\dots,4$ are binary variables that take value 1 if the corresponding pair is active and 0 if it is inactive for a given circuit.

$$
\begin{aligned}
dlacI = &+ Y(1,2)\alpha_{lac}/(1 + K_{lac1}lacI^4)\\
&+ Y(2,2)\alpha_{lac}/(1 + K_{lac2}lacI^4)\\
&+ Y(3,2)\alpha_{lac}/(1 + K_{lac3}lacI^4)\\
&+ Y(4,2)\alpha_{lac}/(1 + K_{lac4}lacI^4)\\
&+ Y(5,2)\alpha_{\lambda}/(1 + K_{lambda}cI^2)\\
&+ Y(6,2)\alpha_{tet}/(1 + K_{tet1}tetR^2)\\
&+ Y(7,2)\alpha_{tet}/(1 + K_{tet2}tetR^2)\\
&+ Y(8,2)\alpha_{ara}/(1 + K_{araC}araC^2)\\
&- Kf \cdot lacI \cdot IPTG\\
&+ Kb \cdot lacIIPTG\\
&- K_{deglacI} \cdot lacI\\
dlacIIPTG = &+Kf \cdot lacI \cdot IPTG\\
&- Kb \cdot lacIIPTG\\
&- K_{degcpx} \cdot lacIIPTG\\
dtetR = &+ Y(1,1)\alpha_{lac}/(1 + K_{lac1}lacI^4)\\
&+ Y(2,1)\alpha_{lac}/(1 + K_{lac2}lacI^4)\\
&+ Y(3,1)\alpha_{lac}/(1 + K_{lac3}lacI^4)\\
&+ Y(4,1)\alpha_{lac}/(1 + K_{lac4}lacI^4)\\
&+ Y(5,1)\alpha_{\lambda}/(1 + K_{lambda} \cdot cI^2)\\
&+ Y(6,1)\alpha_{tet}/(1 + K_{tet1}tetR^2)\\
&+ Y(7,1)\alpha_{tet}/(1 + K_{tet2}tetR^2)
\end{aligned}
$$

$$
\begin{aligned}
&+ Y(8,1)\alpha_{tet}/(1 + K_{araC}araC^2)\\
&- KftetR \cdot aTc\\
&+ Kb\ tetRaTc\\
&- K_{degtetR}tetR\\
dtetRaTc = &+ Kf \cdot tetR \cdot aTc\\
&- Kb \cdot tetRaTc\\
&- K_{degcpx} \cdot tetRaTc\\
dcI = &+ Y(1,3)\alpha_{lac}/(1 + K_{lac1}lacI^4)\\
&+ Y(2,3)\alpha_{lac}/(1 + K_{lac2}lacI^4)\\
&+ Y(3,3)\alpha_{lac}/(1 + K_{lac3}lacI^4)\\
&+ Y(4,3)\alpha_{lac}/(1 + K_{lac4}lacI^4)\\
&+ Y(5,3)\alpha_{\lambda}/(1 + K_{lambda} \cdot cI^2)\\
&+ Y(6,3)\alpha_{tet}/(1 + K_{tet1}tetR^2)\\
&+ Y(7,3)\alpha_{tet}/(1 + K_{tet2}tetR^2)\\
&+ Y(8,3)\alpha_{tet}/(1 + K_{araC}araC^2)\\
&- K_{degcI}cI\\
daraC = &+ Y(1,4)\alpha_{lac}/(1 + K_{lac1}lacI^4)\\
&+ Y(2,4)\alpha_{lac}/(1 + K_{lac2}lacI^4)\\
&+ Y(3,4)\alpha_{lac}/(1 + K_{lac3}lacI^4)\\
&+ Y(4,4)\alpha_{lac}/(1 + K_{lac4}lacI^4)\\
&+ Y(5,4)\alpha_{\lambda}/(1 + K_{lambda} \cdot cI^2)\\
&+ Y(6,4)\alpha_{tet}/(1 + K_{tet1}tetR^2)\\
&+ Y(7,4)\alpha_{tet}/(1 + K_{tet2}tetR^2)\\
&+ Y(8,4)\alpha_{tet}/(1 + K_{araC}araC^2)\\
&- K_{degaraC}araC.
\end{aligned}
$$

The values of the parameters are included in the following Table S1.

Table S1 Parameters from Dasika and Maranas [1]

Parameter	value	units
α_{lac}	1.215	
α_{tet}	1.215	
α_{λ}	2.92	
α_{ara}	1.215	
K_{λ}	0.33	nm^{-2}
K_{tet1}	0.014	nm^{-2}
K_{tet2}	1.4	nm^{-2}
K_{lac1}	10	nm^{-2}
K_{lac2}	0.01	nm^{-2}
K_{lac3}	0.001	nm^{-2}
K_{lac4}	0.00001	nm^{-2}
K_{araC}	2.5	nm^{-2}
$K_{deglacI}$	0.0346	s^{-1}
$K_{degtetR}$	0.0346	s^{-1}
K_{degcI}	0.0693	s^{-1}
$K_{degaraC}$	0.0115	s^{-1}
K_{degcpx}	0.0693	s^{-1}
K_f	0.05	$nm^{-1}s^{-1}$
K_b	0.1	s^{-1}

References

[1] Dasika, M.S., Maranas, C.D.: Optcircuit: An optimization based method for computational design of genetic circuits. BMC Syst Biol 2, 24 (2008)
