# Supplementary material for: Multicriteria global optimization for biocircuit design
Source: BMC Syst Biol. 2014 Sep 24;8:113. doi: 10.1186/s12918-014-0113-3 (PMC4180256; doi:10.1186/s12918-014-0113-3)
Supplement: Additional file 2 — Supplementary Figures. Figures S1, S2, S3, S4 and S5. [file 12918_2014_113_MOESM2_ESM.pdf]

Additional File 2.

Multicriteria global optimization for biocircuit design

Irene Otero-Muras and Julio R. Banga

Bioprocess Engineering Group, Spanish Council for Scientific Research, IIM-CSIC, Eduardo Cabello 6, 36208 Vigo (Spain)

This additional file contains five supplementary figures not included in the main text for space reasons.

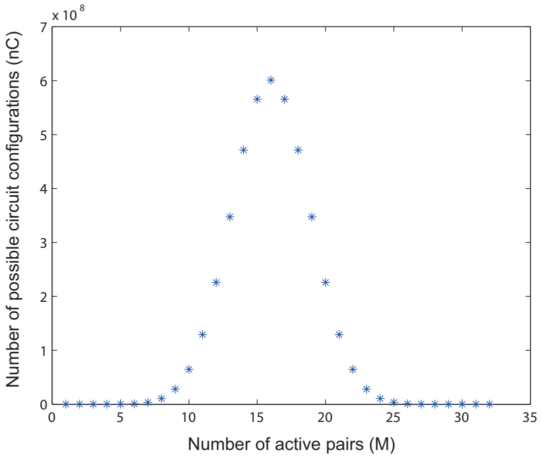

Figure S1 Number of possible circuit configurations as a function of the number of active pairs (for a library with $p = 32$ possible pairs).

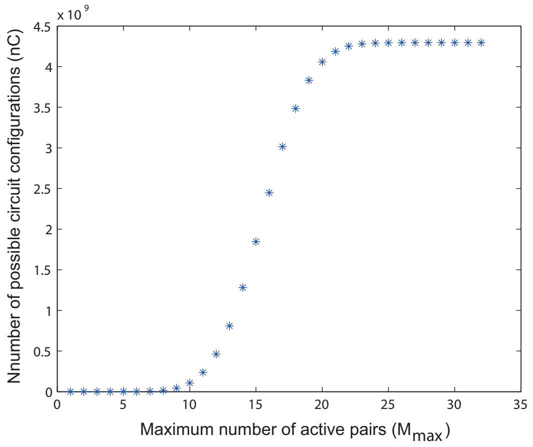

Figure S2 Number of possible circuit configurations for increasing M_{max} (for a library with $p = 32$ possible pairs).

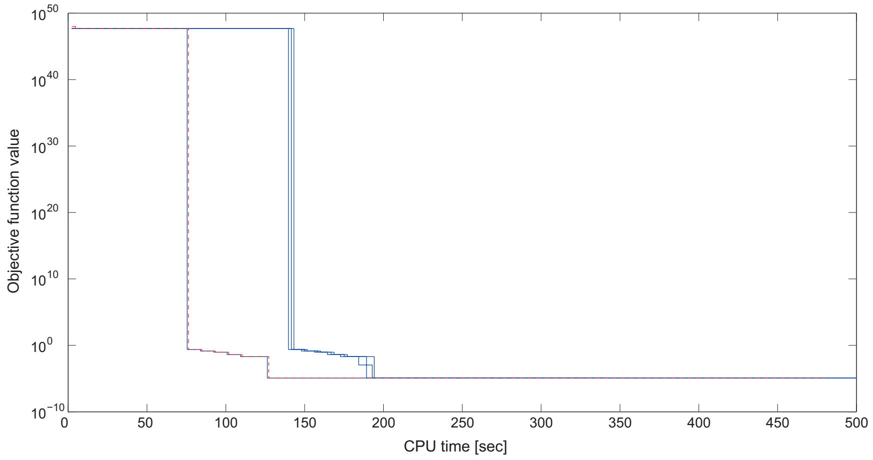

Figure S3 MITS Convergence curves for different initial guesses with $M_{max} = 2$. The initial guess for the red dashed convergence curve is the circuit with no active pairs. To make possible a log scale representation the objective function has been shifted to the positive orthant. Results obtained with Matlab under Windows 7 in a PC Intel 2.8 GHz Xeon.

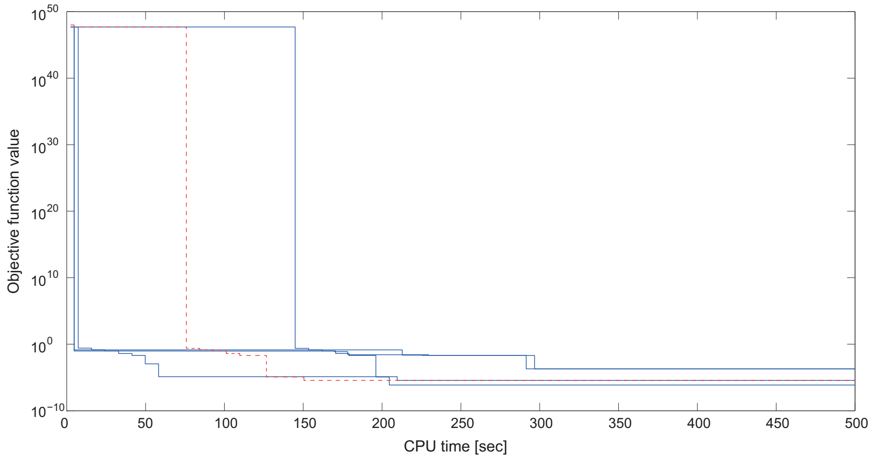

Figure S4 MITS Convergence curves for different initial guesses with $M_{max} = 3$. The initial guess for the red dashed convergence curve is the circuit with no active pairs. To make possible a log scale representation the objective function has been shifted to the positive orthant. Results obtained with Matlab under Windows 7 in a PC Intel 2.8 GHz Xeon.

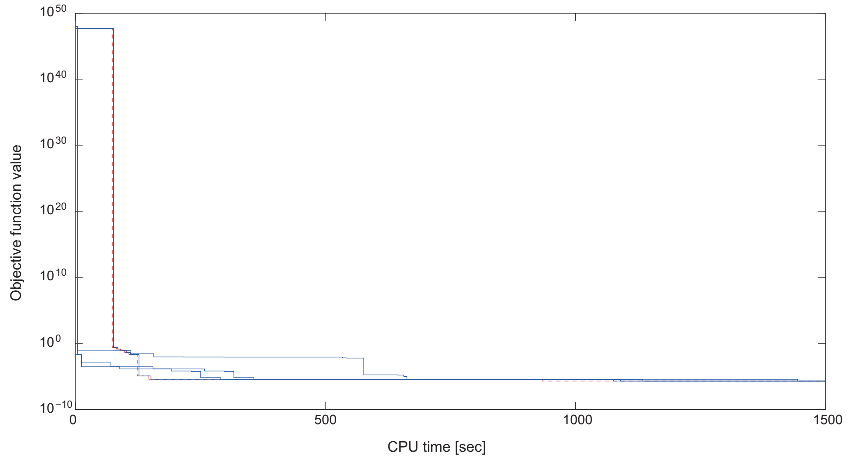

Figure S5 MITS Convergence curves for different initial guesses with $M_{max} = 32$. The initial guess for the red dashed convergence curve is the circuit with no active pairs. To make possible a log scale representation the objective function has been shifted to the positive orthant. Results obtained with Matlab under Windows 7 in a PC Intel 2.8 GHz Xeon.
